# Deciphering Urogenital Cancers through Proteomic Biomarkers: A Systematic Review and Meta-Analysis

**DOI:** 10.3390/cancers16010022

**Published:** 2023-12-20

**Authors:** Aafaque Ahmad Khan, Nahad Al-Mahrouqi, Aida Al-Yahyaee, Hasan Al-Sayegh, Munjid Al-Harthy, Shoaib Al-Zadjali

**Affiliations:** 1 Research Laboratories, Sultan Qaboos Comprehensive Cancer Care and Research Center, Muscat 123, Oman; n.almahrouqi@cccrc.gov.om (N.A.-M.); a.alyahyaee@cccrc.gov.om (A.A.-Y.); h.alsayegh@cccrc.gov.om (H.A.-S.); s.alzadjali@cccrc.gov.om (S.A.-Z.); 2 Medical Oncology Department, Urogenital Cancers Program, Sultan Qaboos Comprehensive Cancer Care and Research Center, Muscat 123, Oman; m.alharthy@cccrc.gov.om

**Keywords:** prostate cancer, bladder cancer, kidney cancer, proteomics biomarkers

## Abstract

**Simple Summary:**

Urogenital cancers (prostate, bladder, kidney) have significant global health impacts. Proteomic biomarkers are emerging tools that aim to improve early detection and personalized treatments. Our study conducted a comprehensive review and meta-analysis of the literature on these biomarkers in plasma, tissue, and urine samples. We found 1879 unique proteins from 37 studies, suggesting their potential as cancer markers. A meta-analysis and pathway analysis revealed their roles in various processes, enhancing our understanding of these cancers. These findings offer promise for improved detection, treatment, and patient outcomes.

**Abstract:**

Urogenital cancers, which include prostate, bladder, and kidney malignancies, exert a substantial impact on global cancer-related morbidity and mortality. Proteomic biomarkers, emerging as valuable tools, aim to enhance early detection, prognostic accuracy, and the development of personalized therapeutic strategies. This study undertook a comprehensive systematic review and meta-analysis of the existing literature investigating the role and potential of proteomic biomarkers in plasma, tissue, and urine samples in urogenital cancers. Our extensive search across several databases identified 1879 differentially expressed proteins from 37 studies, signifying their potential as unique biomarkers for these cancers. A meta-analysis of the significantly differentially expressed proteins was executed, accentuating the findings through visually intuitive volcano plots. A functional enrichment analysis unveiled their significant involvement in diverse biological processes, including signal transduction, immune response, cell communication, and cell growth. A pathway analysis highlighted the participation of key pathways such as the nectin adhesion pathway, TRAIL signaling pathway, and integrin signaling pathways. These findings not only pave the way for future investigations into early detection and targeted therapeutic approaches but also underscore the fundamental role of proteomics in advancing our understanding of the molecular mechanisms underpinning urogenital cancer pathogenesis. Ultimately, these findings hold remarkable potential to significantly enhance patient care and improve clinical outcomes.

## 1. Introduction

Urogenital malignancies, which include prostate, bladder, and kidney cancers, present a significant challenge to global public health systems due to their prevalence and significant morbidity and mortality [1]. Prostate cancer in particular ranks as the second most prevalent cancer among men, with many cases eventually progressing to aggressive stages of disease [2]. Similarly, bladder and kidney cancers feature among the top ten most common cancers, thereby exacerbating the worldwide cancer burden [3]. Although advances in diagnostic techniques and therapeutic interventions have enhanced patient outcomes, these malignancies are frequently diagnosed at advanced stages, attributable to their asymptomatic presentation during early stages and the absence of sensitive, specific screening tools, especially for bladder and kidney cancers [4,5]. Urogenital cancers are characterized by a complex and heterogeneous pathophysiology which can be attributed to a diverse range of genetic and epigenetic alterations [6]. These alterations can impact critical biological pathways, leading to a range of phenotypic changes and clinical outcomes [7]. The identification of biomarkers that can reliably capture the heterogeneity of urogenital cancers and predict their course remains a significant challenge in the field of oncology [7]. In urologic malignancies, Crocetto et al. highlighted the compelling need for effective biomarkers to personalize treatment strategies and improve clinical outcomes and highlighted the importance of ongoing research into biomarkers that can enhance the precision of therapeutic interventions in urogenital cancers [8]. In addition, these malignancies often exhibit resistance to conventional treatment approaches, leading to poor prognosis and reduced survival rates [9]. Therefore, the identification of novel biomarkers that can aid in the early detection, prognosis, and personalized treatment of these cancers is an urgent need.

Proteomics has recently emerged as a powerful analytical tool in advancing our understanding of cancer biology. Proteomic biomarkers, which reflect dynamic physiological and pathological changes in the disease state, hold significant promise for early detection, prognosis, and the development of personalized treatment strategies in urogenital cancers [10,11]. Proteomics has several advantages over traditional approaches, including its ability to provide a comprehensive snapshot of the protein complement of a cell, tissue, or organism at a given time. This offers valuable functional evidence of genome expression and enables the identification of molecular alterations that drive cancer progression [12]. Furthermore, proteomics facilitates the elucidation of signaling pathways and the identification of therapeutic targets. Several recent studies have demonstrated the potential of proteomics in the identification of biomarkers and therapeutic targets in urogenital cancers, highlighting the importance of conducting further research in clinical settings [13,14,15]. While a number of studies on the role of proteomics in urogenital cancers have begun to be published, a comprehensive synthesis and analysis of these findings is lacking. Unanswered questions remain about the consistency of these potential proteomic biomarkers across different studies and their functional significance in disease pathways. Moreover, given the rapid advancement of proteomic technologies and the increasing complexity of generated data, the methodological quality of these studies warrants close scrutiny.

Considering the significance of proteomic biomarkers in urogenital cancers, this systematic review and meta-analysis aims to systematically identify and analyze studies that investigate proteomic biomarkers in urogenital cancers. Our objectives include summarizing the potential biomarkers identified thus far, examining their differential expression in cancerous versus control samples, elucidating their biological significance through functional enrichment and pathway analyses, and evaluating the methodological quality of the included studies using the QUADOMICS tool, a version of QUADAS-2 specifically adapted for omics studies. By conducting this comprehensive analysis, we aim to provide a more coherent understanding of the current state of proteomic biomarker research in urogenital cancers and highlight potential directions for future investigations.

## 2. Materials and Methods

### 2.1. Study Design

This systematic review and meta-analysis aimed to synthesize and analyze the existing literature on proteomic biomarkers in urogenital cancers. This study followed the Preferred Reporting Items for Systematic Reviews and Meta-Analyses (PRISMA) guidelines to ensure transparency and rigor in the conduct and reporting of the review.

### 2.2. Search Strategy and Selection Criteria

A comprehensive search strategy was developed to identify relevant studies published as of May 2023. The following databases were searched: PubMed, MEDLINE, CINAHL Scopus, and any additional relevant sources. The search terms and keywords used included variations of “proteomics” and specific cancer types such as “prostate cancer”, “bladder cancer”, and “kidney cancer”. Studies were included if they met the following criteria: (1) investigated proteomic biomarkers in urogenital cancers, (2) reported on differential protein expression between cancerous and control samples, (3) included human subjects, and (4) were published in English. Studies were excluded if they were reviews, conference abstracts, or did not meet the inclusion criteria.

### 2.3. Data Extraction

Articles from all databases were combined, and duplicates were removed using the JabRef tool (version 5.11). A non-redundant list of articles was screened based on title and abstract for eligible articles. Data were independently extracted from the eligible studies by three authors (AAK, NM, and AY) in a Google spreadsheet. The following information was extracted: study characteristics (e.g., authors, publication year), participant demographics (e.g., sample size, cancer type), proteomic biomarker data (e.g., fold changes, *p*-values), type of proteomics technique used, and other validation techniques, if available.

### 2.4. Quality Assessment

The methodological quality of the included studies was assessed using a modified version of the QUADAS-2 tool specifically adapted for omics studies, known as QUADOMICS. The QUADOMICS tool considers the quality of study design, sample collection and preparation, proteomic profiling methods, and data analysis procedures. Two independent reviewers (NM, AY) assessed the quality of each study, and any discrepancies were resolved through a discussion or consultation with a third reviewer (AAK).

### 2.5. Meta-Analysis

A comprehensive meta-analysis was executed, utilizing the statistical data delineated in the individual studies using the Amanida R-package (version 0.2.3) [16]. This package incorporated both *p*-values and fold-change values from individual studies and employed the Fisher test to combine *p*-values, considering sample sizes in each study. This approach squared *p*-values and compared them to a chi-squared distribution, accounting for their uniform distribution assumption. Weighting by sample size was applied to enhance result reliability. Larger studies carried greater weight, ensuring that proteins exhibiting consistent changes across multiple studies with larger sample sizes were prioritized for a significance assessment. The fold-change values underwent a logarithmic transformation, and a weighted average was calculated contingent on the study’s sample size. In an effort to uphold the directionality of the results, data entries were stratified into categories of upregulated and downregulated entities.

### 2.6. Functional Enrichment and Pathway Analysis

A list of significantly differentially expressed proteins was extracted from the dataset using the following criteria. Firstly, proteins were considered for inclusion if their expression levels were at least two-fold greater in cancer samples compared to control samples. Secondly, the *p*-value associated with protein expression had to be equal to or less than 0.05, indicating statistical significance. In cases in which only one mass-spectrometry-based study supported the differential expression, validation using alternative techniques such as immunohistochemistry (IHC), Western blotting, or an enzyme-linked immunosorbent assay (ELISA) was necessary. To gain further insights into the biological functions and pathways associated with the differentially expressed proteins, functional enrichment and pathway analyses were performed using the Functional Enrichment Analysis (FunRich) tool (version 3.1.4) [17]. An exploration of functional associations among proteins identified through an MS analysis was conducted using the String v. 9.1 database (http://string-db.org/, URL accessed on 10 November 2023). STRING operates as a comprehensive resource compiling available data on protein–protein associations, scoring and weighting them, supplementing with predicted interactions, and extracting results from automated literature-mining searches. The search parameters for a minimum interaction were set to a high confidence level (0.7) with a limit of 10 interactors.

## 3. Results

**Figure 1:**
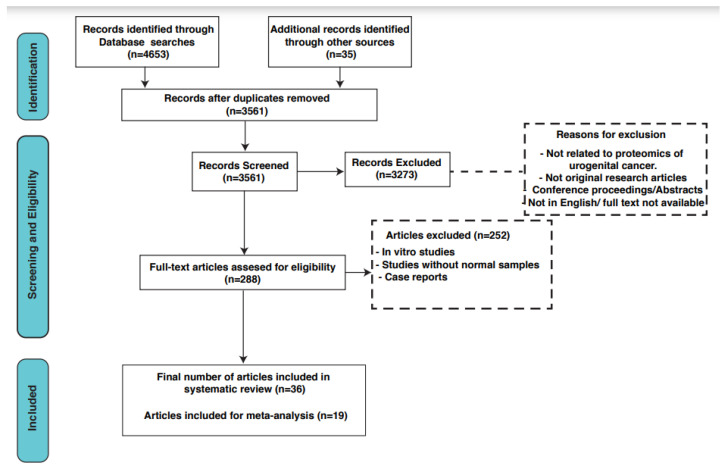
A workflow depicting the systematic search strategy for the scientific literature included in this review.

**Figure 2:**
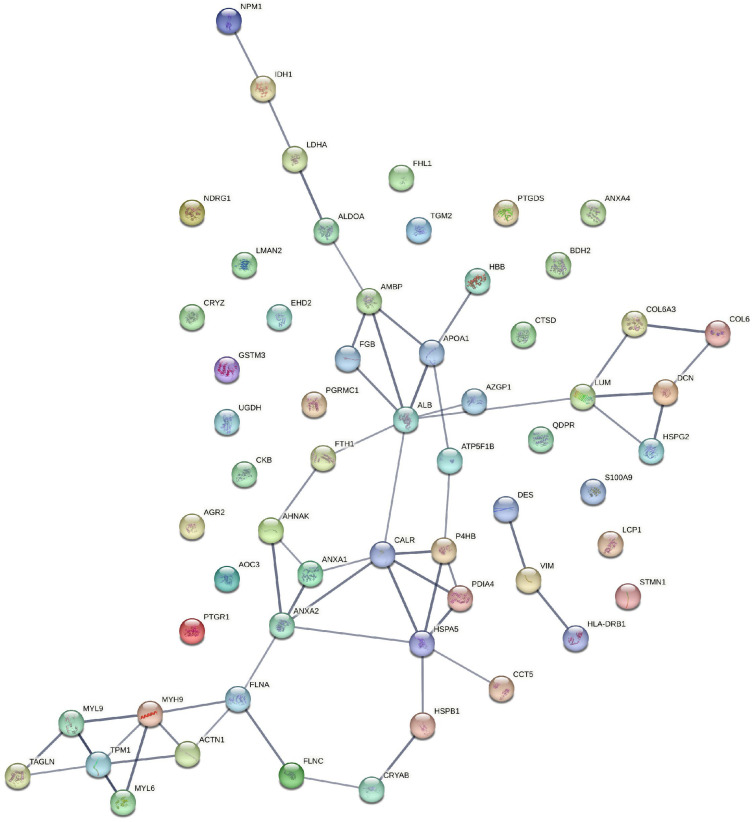
STRING network analysis of commonly differentially expressed proteins in prostate, bladder, and kidney cancers. This network includes 61 commonly differentially expressed proteins in prostate, bladder, and kidney cancers identified in this systematic review. Interacting proteins are connected by lines, and line thickness corresponds to a strong interaction.

A total of 3561 articles were identified after removing duplicates from all databases. After screening these articles based on their titles and abstracts, 288 relevant articles were selected for a full-text review. Finally, based on our inclusion and exclusion criteria, 37 articles met the requirements and were included in this systematic review. A detailed search strategy is depicted in Figure 1. These included articles were comparative studies in which the authors compared urogenital cancer patient samples with control samples. The total sample size of cancer patients was 733, while 993 control samples were included. Various pieces of useful information from the included studies were annotated which included the name and symbol for differentially expressed proteins, cohort information, MS and validation methods, and fold change and *p*-values for differential proteins (Supplementary Table S1). In total, we identified 1879 differentially expressed proteins from all three cancer types, of which 823 were significantly differentially expressed. There were 61 differential proteins that were common between all three cancer types, as shown in Supplementary Figure S1. For these common proteins, an analysis of protein–protein interactions using the STRING network was carried out to recognize proteins that are functionally interconnected (Figure 2). The interaction study included differentially expressed proteins along with 10 additional interactors. The generated network was visualized in a confidence view in which the strength of associations was indicated by the thickness of the lines or edges connecting the nodes, which represented the proteins. Thicker lines suggested stronger associations, and thinner lines indicated weaker ones.

### 3.1. Functional Enrichment Analysis

**Figure 3:**
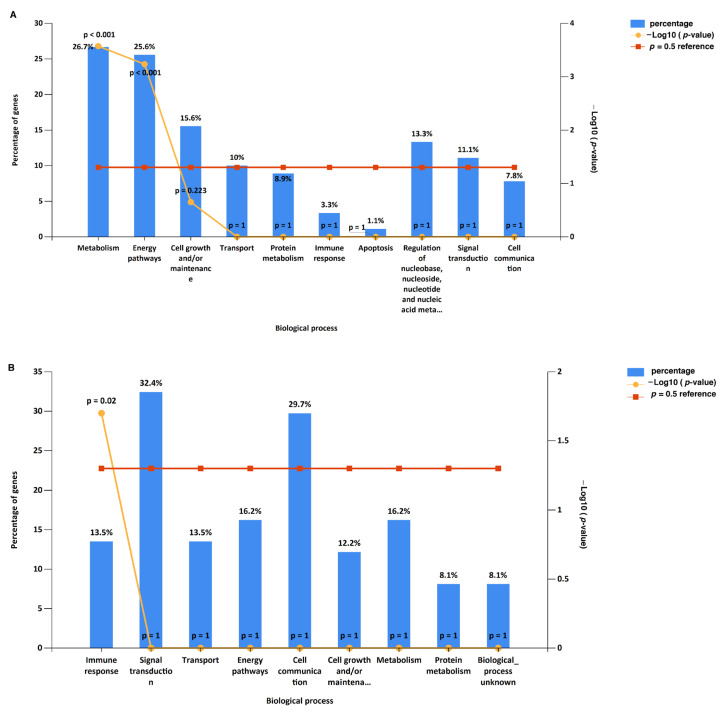

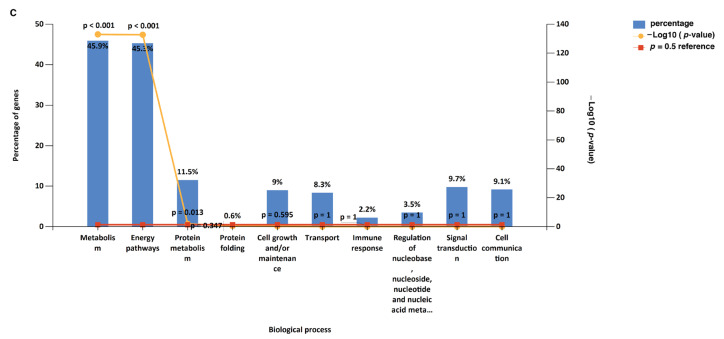
GO biological processes for significantly expressed proteins in prostate, bladder, and kidney cancers: (**A**) 108 proteins from prostate cancer included; (**B**) 143 proteins from bladder cancer included; (**C**) 248 proteins from kidney cancer included.

A gene ontology and pathway enrichment analysis of significantly expressed proteins in prostate, bladder, and kidney cancers was carried out using FunRich. The most enriched biological processes from the significantly expressed proteins in prostate cancer were energy pathways (20.4%), metabolism (20.4%), cell growth and/or maintenance (14.8%), and transport (13%), while in bladder cancer, signal transduction (32.6%), cell communications (30.2%), immune response (20.9%), and transport (14%) were enriched. A kidney cancer data analysis for biological processes showed the enrichment of metabolism (45.9%), energy pathways (45.2%), protein metabolism (11.5%), and cell growth and/or maintenance (9%). Providing a comprehensive overview, the enriched biological processes across all three cancer types are depicted in Figure 3. In prostate cancer, an analysis of significantly expressed proteins revealed the most enriched molecular functions to be related to cytoskeleton I protein binding (9.3%), transporter activity (9.3%), and the structural constituents of cytoskeleton (5.6%). Similarly, in bladder cancer, the enriched molecular functions included calcium ion binding (18.6%), transporter activity (16.3%), and complement activity (9.3%). For kidney cancer, the data analysis highlighted the catalytic activity (18.6%), oxidoreductase activity (7.5%), and hydrolase activity (4.2%). A comprehensive overview of the enriched molecular functions across all three cancer types can be found in Supplementary Figure S2, and cellular localizations of the significantly differentially expressed proteins are depicted in Supplementary Figure S3.

### 3.2. Pathway Analysis

**Figure 4:**
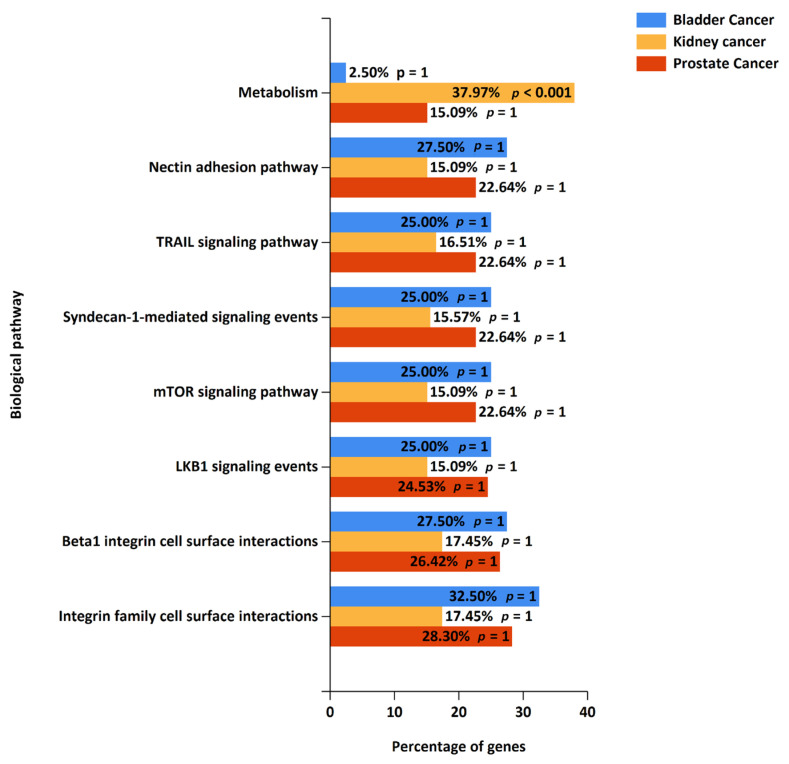
Altered pathways (combined) for significantly expressed proteins in prostate, bladder, and kidney cancers; 108 proteins from prostate cancer, 143 proteins from bladder cancer, and 248 proteins from kidney cancer were included in the analysis.

For a pathway analysis, gene identifiers for significantly expressed proteins from prostate, bladder, and kidney cancer studies were exported into FunRich, and the top 10 enriched biological pathways from each cancer were identified. In prostate cancer, the altered pathways include smooth muscle contraction, muscle contraction, the epithelial-to-mesenchymal transition, semaphorin interactions, endosomal sorting complex required for transport (ESCRT), and membrane trafficking. The top 10 enriched pathways from prostate cancer, along with their *p*-values, are represented in Table 1. Similarly, in bladder cancer, the top enriched biological pathways include the immune system, complement cascade, beta3 integrin cell surface interactions, the mesenchymal-to-epithelial transition, the epithelial-to-mesenchymal transition, and signaling by FGFR (Table 2). In kidney cancer, the enriched pathways include the metabolism of amino acids and derivatives, the metabolism of lipids and lipoproteins, fatty acid, triacylglycerol, and ketone body metabolism, pyruvate metabolism and citric acid (TCA) cycle, fatty acid beta-oxidation, and glucose metabolism (Table 3). A pathway analysis of prostate and bladder cancer proteomic data with stringent false discovery rate (FDR) correction led to the observation that initial pathway enrichments do not maintain statistical significance. This result is indicative of the rigorous nature of FDR correction applied to minimize the false-positive rate, which can also increase the likelihood of type II errors, potentially overlooking biologically pertinent pathways. A combined pathway analysis of significantly expressed proteins from all three cancer types showing common altered pathways is depicted in Figure 4, while individual cancer pathway alterations are shown in Supplementary Figure S4.

### 3.3. Meta-Analysis

**Figure 5:**
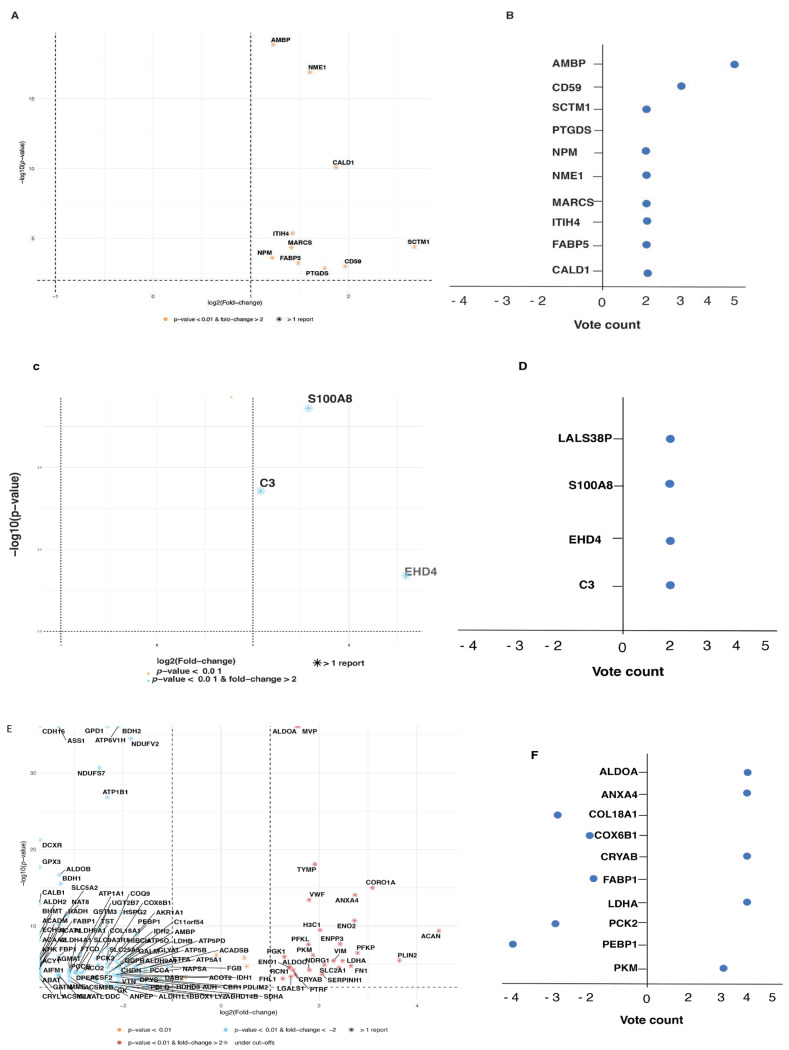
Volcano plots and vote plots for significantly expressed proteins in prostate, bladder, and kidney cancers. (**A**). Volcano plot for proteins from prostate cancer. (**B**). Vote count for proteins from prostate cancer. (**C**). Volcano plot for proteins from bladder cancer. (**D**). Vote count for proteins from bladder cancer. (**E**). Volcano plot for proteins from kidney cancer. (**F**). Vote count for proteins from kidney cancer. Only proteins supported by at least 2 studies were included. Fold change cutoff ≥ 2, and *p*-value ≤ 0.01 was used.

For the meta-analysis, we only included significantly expressed proteins which were supported by at least two studies. A combined *p*-value and fold change value from each study for a particular protein from each cancer type was calculated. For vote counting, a score of +1 was awarded for upregulation, −1 was awarded for downregulation, and 0 was awarded for no change for each study, and the final count is reported. Volcano plots and vote count plots for significantly expressed proteins in each cancer types are depicted in Figure 5A–F. In total, 10 proteins from prostate cancer, 3 proteins from bladder cancer, while 126 proteins from kidney cancer satisfied the criteria, and meta-analysis results for these proteins are summarized in Table 4 (only the top 10 proteins from kidney cancer are depicted, and a complete list is provided in Supplementary Table S2).

### 3.4. Prostate Cancer Biomarkers

We included 11 articles from prostate cancer, reporting 588 proteins in this systematic review. Based on data quality reported, six articles reporting 111 significant differential proteins were used for further data analysis. Multiple protein markers in the prostate cancer samples were assessed, and most of them were elevated compared with normal prostate samples. In one study, Davalieva et al. [18] employed a two-dimensional difference gel electrophoresis (2-D DIGE) mass spectrometry technique for a proteomic analysis of urine samples collected from patients diagnosed with either prostate cancer (PCa) or benign prostatic hyperplasia. They identified a range of diagnostic biomarkers for prostate cancer which included Alpha-1-Microglobulin/Bikunin Precursor (AMBP), Cluster of Differentiation 59 (CD59), Inter-alpha-trypsin Inhibitor Heavy Chain 4 (ITIH4), Prostaglandin D2 Synthase (PTGDS), Secreted and Transmembrane 1 (SCTM1), and Haptoglobin (HP). In the evaluation of various biomarker synergies, a superior degree of diagnostic accuracy was demonstrated by the tandem of Haptoglobin (HP) and Alpha-1-Microglobulin/Bikunin Precursor (AMBP), outperforming the traditional prostate-specific antigen (PSA) marker. This selected suite of biomarkers, noted for its cost-effectiveness and expeditious quantification capabilities, offers promising potential for augmenting both the detection sensitivity and specificity associated with prostate cancer diagnosis [18]. Similarly, Fujita et al. also reported differentially expressed proteins from extracellular vesicles (EVs) from prostate cancer patients’ urine samples [19]. Among them, fatty acid binding protein 5 (FABP5), Alpha-1-Microglobulin/Bikunin Precursor (AMBP), Charged Multivesicular Body Protein 4A (CHMP4A), and Charged Multivesicular Body Protein 4C (CHMP4C) were significantly differentially expressed in prostate cancer patients compared to normal samples [19]. Another study by Webber et al. compared the protein profiles of healthy and disease-affected stroma extracted from patients diagnosed with prostate cancer in order to identify the distinguishing characteristics inherent to stroma that are associated with the disease state [20]. They identified several differentially expressed proteins using a MALDI-TOF analysis including Caldesmon 1 (CALD1), Myristoylated Alanine-Rich C-Kinase Substrate (MARCKS), and Nucleophosmin (NPM), which were significantly expressed [20]. In a study, Frantzi et al. investigated urine samples from 823 patients with prostate-specific antigen (PSA) levels below 15 ng/mL which were collected pre-biopsy. The analysis involved a training set of 543 patients and a validation set of 280 patients [21]. The independent validation of a 19-biomarker (PPP1R3A, F5GYX3, X3CL1, and several collagen family members) model showed a 90% sensitivity, 59% specificity, and an area under the curve (AUC) of 0.81, outperforming both PSA (AUC = 0.58) and the ERSPC-3/4 risk calculator (AUC = 0.69) [21]. Another study by Iglesias-Gato et al. used 28 tumor and 8 normal FFPE tissues from prostate cancer patients and used a SILAC-based LC/MS quantitative proteomics technique to identify differentially expressed proteins [22]. Proteins including carnitine palmitoyl transferase 2 (CPT2, involved in fatty acid transport), coatomer protein complex subunit alpha (COPA, associated with vesicle secretion), and mitogen- and stress-activated protein kinases 1 and 2 (MSK1/2, protein kinases), all of which are overexpressed in tumor cells, contribute to the regulation of prostate cancer (PCa) cell proliferation. In addition, an overexpression of pro-neuropeptide Y (pro-NPY) was detected in PCa (a five-fold increase, *p* < 0.05), with its presence predominantly absent in other types of solid tumors [22]. Another study by Jiang et al. used 2D-DIGE MS for the identification of differentially expressed proteins in pancreatic cancer tissues and reported 60 proteins [23]. Based on a network analysis, they selected PTEN, SFPQ, and HDAC1 for exploring their clinical significance. Intriguingly, the protein PTEN has been identified as an autonomous prognostic indicator for the biochemical recurrence-free survival in patients with prostate cancer (PCa) [23].

### 3.5. Bladder Cancer Biomarkers

We included 11 articles from bladder cancer reporting 813 protein markers. Six articles reporting 143 significantly differentially expressed proteins were included for further analysis. Nedjadi et al. employed 2D-DIGE and mass spectrometry techniques to identify circulating plasma proteins which are differentially expressed in bladder cancer patients compared to normal samples [24]. They reported 15 significantly expressed proteins which include Complement components C3 and C6, haptoglobin (HP), Kelch domain-containing protein 8B (KLHDC8B), Complement C1r subcomponent (C1R), Ceruloplasmin (CP), Immediate early response 3-inter-acting protein1 (IER3IP1), and Integrator complex subunit 10 (INTS10). Interestingly, haptoglobin effectively differentiated low-grade bladder cancer patients from controls with high accuracy (AUC > 0.87), suggesting its potential as a biomarker for early bladder cancer detection, pending further validation [24]. Another study by Bansal et al. employed MALDI-TOF MS to identify differentially expressed proteins in low-grade/high-grade bladder cancer samples compared to normal bladder samples [25]. Using an MS-based discovery approach combined with a Western blotting (WB)/ELISA-based validation approach, they reported five differentially expressed proteins including S100A4, S100A8, S100A9, carbonic anhydrase I, and annexin V. Furthermore, two biomarkers, S100A8 and S100A9, demonstrated high accuracy (ROC, 0.946) in differentiating 81% of bladder cancer (both low-grade and high-grade) cases from healthy controls, exhibiting the highest levels of sensitivity and specificity. Similarly, using a comparable approach, the biomarkers S100A8 and S100A4 accurately (ROC, 0.941) distinguished 92% of low-grade cases from high-grade ones, with the utmost levels of sensitivity and specificity [25]. Another study by Gómez et al. used serum samples to compare two NMIBC subtypes, T1 and Ta, with normal samples using the SWATH-MS technique and identified 40 differentially expressed proteins [26]. Important altered proteins implicated in the complement and coagulation cascade pathways and apolipoproteins include Galectin-3-binding protein (LGALS3BP), Alpha-1-antitrypsin (SERPINA1), Alpha-1-antichymotrypsin (SERPINA3), Apolipoprotein A-II (APOA2), Hemoglobin subunit beta (HBB), and Apolipoprotein F (APOF) [26]. In a similar vein, Lee et al. reported 56 proteins that were significantly differently expressed in extracellular vesicles derived from the urine of bladder cancer patients when contrasted with normal samples [27]. These differential proteins include Guanine nucleotide-binding protein subunit alpha-11 (GNA11), EH domain-containing protein 4 (EHD4), Annexin A1 (ANXA1), Gamma-glutamyl hydrolase (GGH), Gelsolin (GELS), Epidermal growth factor receptor kinase substrate 8 (EPS8), Deoxyribonuclease-1 (DNAS1), and Uromodulin (UROM) [27]. Another study by Sathe et al. used urine samples from muscle-invasive (MIBC) and non-muscle-invasive bladder cancer (NMIBC) patients along with normal controls for a quantitative proteomics analysis [28]. They identified several key proteins that are altered between these patients which include fibrinogen alpha chain isoform alpha-E preproprotein (FGA), laminin subunit alpha-4 isoform X1 (LAMA4), vesicular integral-membrane protein VIP36 precursor (LMAN2), protein S100-A8 isoform a (S100A8), protein S100-A9 (S100A9), and plastin-2 (LCP1). They also detected unique N-glycosylation patterns of CD44, MGAM, and GINM1 that varied between non-muscle-invasive bladder cancer (NMIBC) and muscle-invasive bladder cancer (MIBC) patients which could potentially be linked with the progression of bladder cancer [28]. In one study, Smalley et al. identified eight significantly differentially expressed proteins in the urine microparticles of bladder cancer patients using an LC-MS/MS technique [29]. These include five proteins from the EGFR pathway (EGFR, NRAS, EPS8L2, EPS8L1, and EHD4) and the alpha subunit of GsGTP binding protein, resistin, and retinoic-acid-induced protein 3 [29].

### 3.6. Kidney Cancer Biomarkers

We included 14 qualified articles from kidney cancer representing 884 differentially expressed proteins. We selected seven articles which reported 248 significantly differentially expressed proteins for further analysis. In one study, Song et al. employed a DIA-based LC-MS/MS strategy to examine clear cell renal cell carcinoma patients’ tissues and normal samples and identified 436 differentially expressed proteins [30]. Among the differentially expressed proteins, notable upregulated proteins include L-lactate dehydrogenase A chain (LDHA), annexin A4 (ANXA4), nicotinamide N-methyltransferase (NNMT), gamma-enolase (ENO2), ATP-dependent 6-phosphofructokinase (PFKP), perilipin-2 (PLIN2), protein-glutamine gamma-glutamyl transferase 2 (TGM2), and the expression levels of L-lactate dehydrogenase A chain, annexin A4,nicotinamide N-methyltransferase, and perilipin-2 were further validated using RT-qPCR, Western blot, and immunohistochemistry, which confirmed the findings of the proteomics data [30]. Similarly, Garibaldi et al. employed 2DE-MALDI TOF mass spectrometry for a proteomics analysis of neoplastic and healthy control tissues of renal cell carcinoma patients and identified 18 differentially expressed proteins [31]. Notably upregulated proteins included reticulocalbin-1 (RCN1), alpha-enolase (ENO1), phosphoglycerate kinase 1 (PGK1), retinol-binding protein 4 (RBP4), alpha-crystallin B chain (CRYAB), and triosephosphate isomerase (TPI1). Among these proteins, RCN1 was upregulated in all cancer specimens analyzed via proteomics and its expression was further validated using Western blotting and immunohistochemistry, which suggested it as a potential biomarker for renal cell carcinoma [31]. Another study by Koch et al. used fresh and FFPE samples from renal cell carcinoma patients (both tumor and adjacent normal) and reported over 1000 differentially expressed proteins [32]. The most significant proteins included aggrecan core protein (ACAN), perilipin-2 (PLIN2), solute carrier family 2 (SLC2A1), monocarboxylate transporter 4 (SLC16A3), minor histocompatibility protein HA-1 (HMHA1), blood group Rh(CE) polypeptide (RHCE), dedicator of cytokinesis protein 2 (DOCK), and 2′-5′-Oligoadenylate synthase 2 (OAS2) [32]. A similar study by Atrih et al. employed a quantitative proteomics technique to identify differentially expressed proteins from fresh-frozen tumors and adjacent normal tissues from renal cell carcinoma patients [33]. They identified 596 differentially expressed proteins which included Von Willebrand factor (VWF), ectonucleotide pyrophosphatase/phosphodiesterase family member 3 (ENPP3), histone H3.1 (H3C1), thymidine phosphorylase (TYMP), aggrecan core protein (ACAN), coronin 1A (CORO1A), and ADFP protein (ADFP). The expression levels of CORO1A and ADFP were further validated using immunohistochemistry [33]. Another study by Okamura et al. employed a MALDI-TOF MS technique to profile differential proteins in renal cell carcinoma tissues with respect to adjacent normal samples [34]. They reported several significantly expressed proteins including 6-Phosphofructokinase type C (PFKP), 6-Phosphofructokinase, liver type (PFKL), alpha-Crystallin B chain (CRYAB), alpha-Enolase (ENO1), annexin A4 (ANXA4), apolipoprotein A-I precursor (APOA1), fibrinogen gamma chain precursor (FGG), fibronectin precursor (FN1), four and a half LIM domains protein 1 (FHL1), fructose-bisphosphate aldolase A (ALDOA), galectin-1 (LGALS1), and carnosine dipeptidase 2 (CNDP2). Notably, the expression levels of LGALS1 and CNDP2 were further validated using an RT-PCR and Western blotting [34]. A similar study by Perroud et al. used MALDI-TOF MS to profile protein expression from renal cell carcinoma tissue samples compared to adjacent normal samples [35]. They identified 31 significantly differentially expressed proteins which included serpin H1 precursor (SERPINH1), fructose-bisphosphate aldolase A (ALDOA), vimentin (VIM), apoptosis-inducing factor 1 (AIFM1), glutathione peroxidase 3 precursor (GPX3), aldehyde dehydrogenase (ALDH2), collagen, type XVIII, α-1 (COL18A1), heparan sulfate proteoglycan core precursor (HSPG2), medium chain-specific acyl-CoA dehydrogenase (ACADM), fructose-1,6-bisphosphatase 1 (FBP1), 3-Ketoacyl-CoA thiolase (ACAA2), and phosphatidylethanolamine-binding protein 1 (PEBP1). Notably, the expression levels of serpin H1 precursor (SERPINH1), fructose-bisphosphate aldolase A (ALDOA), vimentin (VIM), and apoptosis-inducing factor 1 (AIFM1) were further validated using immunoblotting and immunohistochemistry [35]. Another study by Weißer et al. reported differential protein expression in FFPE samples from renal cell carcinoma and adjacent normal tissues [36]. They identified 199 differentially expressed proteins which included L-lactate dehydrogenase A chain (LDHA), alpha-crystallin B chain (CRYAB), fructose-bisphosphate aldolase A(ALDOA), annexin A4 (ANXA4), fructose-bisphosphate aldolase C (ALDOC), pyruvate kinase (PKM), stathmin (STMN1), histone H3.1 (H3C1), ATP-dependent 6-phosphofructokinase (PFKP), major vault protein (MVP), perilipin-2 (PLIN2), and Cadherin-16 (CDH16) [36].

**Table 4:** List of proteins from prostate, bladder, and kidney cancers included in meta-analysis. Combined statistics for these proteins are also depicted.

Protein Name	Cancer Type	Expression Trend	Combined and Weighted *p*-Value	Combined and Weighted Fold Change	N_Total	Reference
AMBP	Prostate	Up	1.35339 × 10^−19^	2.34	114	Fujita et al., 2017 [19]; Davalieva et al., 2015 [18]; Davalieva et al., 2015 [18]; Davalieva et al., 2015 [18]; Davalieva et al., 2015 [18]
CALD1	Prostate	Up	8.32538 × 10^−11^	3.66	12	Webber et al., 2016 [20]; Webber et al., 2016 [20]
CD59	Prostate	Up	0.001010766	3.91	72	Davalieva et al., 2015 [18]; Davalieva et al., 2015 [18]; Davalieva et al., 2015 [18]
FABP5	Prostate	Up	0.000586891	2.80	70	Fujita et al., 2017 [19]; Davalieva et al., 2015 [37]
ITIH4	Prostate	Up	4.26138 × 10^−6^	2.69	48	Davalieva et al., 2015 [18]; Davalieva et al., 2015 [18]
MARCS	Prostate	Up	4.4686 × 10^−5^	2.67	12	Webber et al., 2016 [20]; Webber et al., 2016 [20]
NME1	Prostate	Up	1.2542 × 10^−17^	3.04	60	Davalieva et al., 2015 [37]; Jiang et al., 2013
NPM	Prostate	Up	0.000242959	2.33	12	Webber et al., 2016 [20]; Webber et al., 2016 [20]
PTGDS	Prostate	Up	0.001369911	3.38	48	Davalieva et al., 2015 [18]; Davalieva et al., 2015 [18]
SCTM1	Prostate	Up	4.10108 × 10^−5^	6.38	48	Davalieva et al., 2015 [18]; Davalieva et al., 2015 [18]
C3	Bladder	Up	0.001339534	2.07	17	Nedjadi et al., 2020 [24]; Sathe et al., 2020 [28]
EHD4	Bladder	Up	0.004667281	5.99	29	Smalley et al., 2007 [29]; Lee et al., 2018 [27]
LGALS3BP	Bladder	Up	0	1.71	93	Gómez et al., 2021; Smalley et al., 2007 [29]
S100A8	Bladder	Up	0.000434476	2.98	99	Sathe et al., 2020 [28]; Bansal et al., 2014 [25]
ALDOA	Kidney	Up	0	2.903563795	72	Song et al., 2017 [30]; Okamura et al., 2008 [34]; WeiBer et al., 2015 [36]; Perroud et al., 2009 [35]
ANXA4	Kidney	Up	8.87564 × 10^−15^	6.63931328	72	Song et al., 2017 [30]; Okamura et al., 2008 [34]; WeiBer et al., 2015 [36]
COL18A1	Kidney	Down	6.83113 × 10^−8^	0.2	64	Song et al., 2017 [30]; Okamura et al., 2008 [34]; Perroud et al., 2009 [35]
COX6B1	Kidney	Down	2.04982 × 10^−6^	0.2	56	Song et al., 2017 [30]; Okamura et al., 2008 [34]
CRYAB	Kidney	Up	5.68212 × 10^−5^	2.765541687	84	Okamura et al., 2008 [34]; WeiBer et al., 2015 [36]; Giribaldi et al., 2013; Giribaldi et al., 2013 [31]
FABP1	Kidney	Down	3.10267 × 10^−5^	0.1	36	Song et al., 2017 [30]; WeiBer et al., 2015 [36]
LDHA	Kidney	Up	3.56554 × 10^−6^	5.580626486	72	Song et al., 2017 [30]; Okamura et al., 2008 [34]; WeiBer et al., 2015 [36]; Perroud et al., 2009 [35]
PCK2	Kidney	Down	1.13664 × 10^−5^	0.176347496	64	Song et al., 2017 [30]; Okamura et al., 2008 [34]; WeiBer et al., 2015 [36]
PEBP1	Kidney	Down	1.56604 × 10^−9^	0.302889369	112	Song et al., 2017 [30]; Okamura et al., 2008 [34]; Perroud et al., 2009 [35]; Giribaldi et al., 2013 [35]
PKM	Kidney	Up	6.44029 × 10^−7^	3.672247556	64	Song et al., 2017 [30]; Okamura et al., 2008 [34]; WeiBer et al., 2015 [36]

## 4. Discussion

In this systematic review, we compiled and analyzed the current literature related to proteomic biomarkers associated with urogenital cancers, including prostate, bladder, and kidney cancers. Additionally, we performed a meta-analysis of significant biomarkers in these cancers. The results suggest that various proteins exhibit differential expression in cancerous samples compared to healthy controls, highlighting their potential as diagnostic, prognostic, or therapeutic biomarkers. The robustness of this systematic review is reinforced by a well-orchestrated search strategy, predetermined review guidelines, and a rigorous methodology. These approaches enabled us to examine and condense an expansive and seemingly disjointed body of literature. We effectively connected different parts of this research area by using pathway analyses, identifying possible targets for future studies.

In prostate cancer, several biomarkers from different sample types including plasma/serum, tissues, and urine were combined from different studies. In a comprehensive analysis, Larkin et al. utilized isobaric stable isotope labeling (iTRAQ) in conjunction with liquid chromatography–tandem mass spectrometry (LC-MS/MS) to investigate whole, non-depleted serum from various groups, including controls (PSA < 1 ng/mL), benign prostate diseases (such as prostatitis and BPH), early-stage (T1-T2) prostate cancer, and late-stage (T3-T4) cancer [38]. The study identified a total of 1034 proteins, of which 40 were highlighted as the most significantly differentially expressed, exhibiting minimal variability across replicates within the same group. A subset of seven proteins (KLK3, SAA, TSR1, VWA5B2, CST3, SRC, and SGCd) were chosen for additional validation using an enzyme-linked immunosorbent assay (ELISA) based on their distinct capacity to discriminate between the groups. The validation was conducted in an independent cohort, thereby enhancing the robustness of the findings by mitigating potential bias from using the same patient samples for discovery proteomics. Notably, the pre-rRNA-processing protein TSR1 homolog (TSR1), when used in conjunction with PSA, proved to be superior to PSA alone in terms of cancer categorization. The functional enrichment analysis further revealed an overrepresentation of proteins associated with extracellular vesicular exosomes among the differentially expressed proteins, emphasizing their potential role in the disease process [38]. Our meta-analysis of prostate cancer studies identified Alpha-1-microglobulin/bikunin precursor (AMBP) as one of the potential biomarkers, and its role has been explored in various studies, providing significant insights. In a study by Fujita et al. [19], the proteomic profile of prostate cancer was evaluated, and AMBP was identified as one of the proteins that were differentially expressed. This study provided valuable insights into how AMBP could contribute to the tumorigenesis of prostate cancer, adding to the evidence of AMBP’s potential as a diagnostic and prognostic marker [19]. Furthermore, Davalieva et al. [37] performed a comprehensive analysis to investigate the proteome of prostate cancer patients. Their findings emphasized the relevance of AMBP as a biomarker, showing a significant difference in its expression between prostate cancer and benign samples. This supported previous observations about the downregulation of AMBP in prostate cancer tissue and its potential involvement in disease development and progression. Earlier studies, such as those by M’Koma et al. [39] and Ummanni et al. [40], had already indicated the importance of AMBP in prostate cancer. Together with the subsequent findings by Fujita et al. and Davalieva et al., a compelling picture has emerged of AMBP’s role in prostate cancer. The cumulative evidence suggests that AMBP’s differential expression and involvement in various biological processes, including immune response modulation, might have direct implications in prostate cancer pathogenesis. Similarly, in our meta-analysis, fatty acid-binding protein 5 (FABP5) emerged as a key player in prostate cancer and has been explored as a potential biomarker [19,37]. Fujita et al. identified FABP5 among the differentially expressed proteins in urinary extracellular vesicles from high-Gleason-score prostate cancer. The study’s results indicated that FABP5 might be involved in molecular mechanisms related to the aggressiveness and progression of the disease [19]. In addition, Davalieva et al. examined the proteome of prostate cancer patients, finding a significant difference in the expression of FABP5 between cancerous and benign samples. Their findings further support the potential of FABP5 as a diagnostic and prognostic marker in prostate cancer [37]. Other studies, including those by Adamson et al. [41] and Morgan et al. [42], have also shown an association of FABP5 with prostate cancer, particularly with its invasion and migration capabilities. These studies suggest the significant potential of FABP5 as a biomarker in prostate cancer. Its differential expression and correlation with cancer progression warrant further investigations, which may pave the way for more precise diagnostics and targeted therapeutic approaches for prostate cancer patients. Another biomarker in our meta-analysis called NME1, also known as non-metastatic cells 1 protein, has been examined for its potential role as a biomarker in prostate cancer. Additionally, Jiang et al. identified NME1 as one of the significant proteins involved in prostate cancer metastasis [23]. The study’s findings suggest that the downregulation of NME1 may be associated with advanced prostate cancer and might contribute to the aggressive behavior of the disease [23]. In line with this, an earlier study by Steeg et al. also recognized NME1 as a metastasis suppressor gene, and its expression levels have been inversely correlated with the metastatic potential of several types of tumors, including prostate cancer [43]. Additionally, Davalieva et al. conducted a comprehensive proteomics analysis of malignant and benign prostate tissues, finding an altered expression of NME1 in prostate cancer samples [37]. Their investigation indicated that NME1 could be used as a diagnostic and prognostic indicator, adding further support to its relevance in understanding prostate cancer progression. A similar finding was also reported by Marino et al., who focused on the prognostic value of NME1, analyzing its expression levels in prostate cancer tissues and correlating them with clinical outcomes [44]. These studies on NME1 suggest that its expression may be linked to the invasive and metastatic characteristics of prostate cancer, making it a potential biomarker for prostate cancer. These identified biomarkers in prostate cancer have the potential to play a crucial role in shaping personalized treatment strategies, as exemplified by recent research combining the prostate health index (PHI) and multiparametric magnetic resonance imaging (mpMRI) to guide personalized therapy decisions for prostate cancer patients [45].

Similarly, in bladder cancer, we identified several protein markers that are differentially expressed between cancer and control samples. Complement component C3, a pivotal protein in the immune system, has gained attention as a potential biomarker in bladder cancer due to its involvement in the inflammatory response and its association with cancer-related processes. Studies have demonstrated altered expression of C3 in bladder cancer patients, indicating its potential diagnostic and prognostic significance. Nedjadi et al. performed a comprehensive analysis of circulating plasma proteins in bladder cancer patients, identifying complement component C3 among the differentially expressed proteins, thus suggesting its potential as a diagnostic marker [24]. Furthermore, Sathe et al. conducted a quantitative proteomics analysis on urine samples from muscle-invasive and non-muscle-invasive bladder cancer patients, revealing the differential expression of C3 between these patient groups, emphasizing its role as a potential biomarker for disease categorization [28]. Other studies have also implicated C3 in the context of bladder cancer, highlighting its association with immune-related processes and potential clinical implications [46]. Another biomarker, EHD4, a member of the Eps15 homology domain-containing protein family, has garnered attention as a potential biomarker in bladder cancer. Smalley et al. utilized LC-MS/MS to identify significantly differentially expressed proteins in the urine microparticles of bladder cancer patients, in which EHD4 emerged as one of the candidates [29]. Additionally, Lee et al. investigated extracellular vesicles derived from the urine samples of bladder cancer patients using a comparable approach [27]. These studies collectively suggest that EHD4 may play a role in the molecular landscape of bladder cancer. Owing to the genetic and epigenetic diversity within urogenital tumors, the use of single biomarkers may not yield conclusive patient stratification. Thus, the emphasis has shifted toward the use of multiple biomarker panels as opposed to individual markers. Illustratively, in a study by Abogunrin et al., a total of 22 biomarkers were examined in the urine of 80 bladder cancer patients and 77 controls, leveraging ELISA and biochip arrays [47]. The study identified markers with altered expression, such as BTA and NUMA1, showing ROC AUC values in the range of 0.7–0.8. Incorporating demographic variables like age and smoking history into algorithms with these markers bolstered the receiver operating characteristic (ROC) area under the curve (AUC) to 0.9 [47]. This approach is in line with the increasing recognition of the heterogeneity of tumors at the genetic and epigenetic levels. Multi-plex biomarker panels have the potential to provide more accurate and reliable diagnosis, prognosis, and treatment of cancer. However, further studies are needed to validate the proposed biomarker panels in independent cohorts and to evaluate their diagnostic potential in different types of cancer.

In kidney cancer, our analysis identified a relatively large number of biomarkers compared to prostate and bladder cancer. Our meta-analysis identified 126 biomarkers from kidney cancer that are significantly altered with respect to control samples. Song et al. employed an LC-MS/MS-based proteomics analysis in 14 clear cell renal cell carcinoma (ccRCC) patient tissues and adjacent normal samples [30]. They identified 436 dysregulated proteins in cancer samples compared to normal samples. Notably, L-lactate dehydrogenase A chain, annexin A4, nicotinamide N-methyltransferase, and perilipin-2 levels were further validated using RT-qPCR, Western blotting, and immunohistochemistry [30]. Annexin A4 (ANXA4), a calcium-regulated phospholipid-binding protein, has emerged as a potential biomarker candidate in kidney cancer. Song et al. identified ANXA4 as significantly upregulated in kidney cancer tissues through a quantitative proteomics analysis, suggesting its potential involvement in disease pathogenesis [30]. Additionally, Okamura et al. reported ANXA4 as part of the protein signature that distinguishes between kidney cancer and healthy control tissues [34]. WeiBer et al. demonstrated ANXA4 as one of the differentially expressed proteins in the urinary exosomes of kidney cancer patients, indicating its potential as a non-invasive urinary biomarker [36]. Masui et al. reported ANXA4 as a component of the protein profile that characterizes kidney cancer subtypes [33]. These findings collectively highlight the potential of ANXA4 as a biomarker for kidney cancer detection, risk stratification, and monitoring. Nevertheless, further validation studies involving larger patient cohorts are essential to establish its clinical utility. Lactate dehydrogenase A (LDHA), a key enzyme in glycolysis, has been studied as a potential biomarker in kidney cancer. Song et al. identified LDHA as significantly upregulated in kidney cancer tissues through a quantitative proteomics analysis, implying its potential role in disease progression [30]. Moreover, Okamura et al. reported LDHA as part of the protein signature distinguishing kidney cancer from healthy controls in urinary exosomes [34]. Similarly, WeiBer et al. demonstrated LDHA as one of the differentially expressed proteins in the urinary exosomes of kidney cancer patients, highlighting its potential as a non-invasive urinary biomarker [36]. Other studies showed that serum LDH levels are associated with the outcomes of renal cell carcinoma (RCC) and can be used as a valuable biomarker for monitoring progress [48]. Moreover, Wang et al. showed that pre-treatment serum LDH is a significant prognostic factor in high-risk patients with metastatic RCC [49]. Overall, the search results suggest that LDHA may be a useful biomarker for predicting the prognosis and monitoring the progress of kidney cancer, particularly RCC. Other significant biomarkers that are differentially expressed in kidney cancer include Aldolase A, Fructose-Bisphosphate (ALDOA), Collagen Type XVIII Alpha 1 Chain (COL18A1), Cytochrome C Oxidase Subunit 6B1 (COX6B1), Alpha-B Crystallin (CRYAB), Fatty Acid-Binding Protein 1 (FABP1), Phosphoenolpyruvate Carboxykinase 2, Mitochondrial (PCK2), Phosphatidylethanolamine-Binding Protein 1 (PEBP1), and Pyruvate Kinase Muscle (PKM).

When it comes to urological cancers, the balance between the benefits and potential drawbacks of new biomarkers must be taken into account. For instance, biomarkers enabling early prostate cancer detection need to be evaluated against the risk of potential overtreatment. Hence, it is crucial to pair these biomarkers with others that can determine patients who truly require therapeutic intervention. Similarly, the demand for biomarkers exhibiting high sensitivity and specificity for bladder cancer in the context of mass screening does not align with the need for biomarkers used in categorizing patients presenting with hematuria [50]. This is primarily because the latter situation has a comparatively lower disease prevalence, and unnecessary expensive investigations should be avoided. Consequently, merging studies on clinical effectiveness with cost-effectiveness analyses is vital. This approach has been exemplified in the detection and monitoring of bladder cancer using photodynamic diagnosis, cystoscopy, and several urinary biomarkers [50].

Despite its usefulness, our systematic review on proteomics biomarkers of urogenital cancers does carry certain limitations. The inherent heterogeneity in experimental methodologies across the considered studies can result in variances in the identified biomarkers, adding a level of complexity to the synthesis of findings. Moreover, there may exist a potential for publication bias, considering that studies yielding significant results have a higher likelihood of being published. Additionally, the review’s conclusions are contingent upon the quality and completeness of the original studies; hence, any limitations within them directly affect our findings. Furthermore, our conclusions require cautious interpretation due to several factors. Firstly, there is inherent inter-individual variability in biomarker expression, which can impact the reliability of these markers across different patient populations. Additionally, while our analysis identified a compelling list of differentially expressed proteins in urogenital cancers, it is essential to recognize that many of these biomarkers require further validation. This validation process should ideally involve larger and more diverse independent cohorts to confirm their clinical utility. We acknowledge that our study serves as a crucial starting point for identifying potential biomarkers, but their ultimate translation into clinical practice will necessitate rigorous individualized investigations to establish their specific roles in cancer development, diagnosis, prognosis, and treatment.

## 5. Conclusions

This systematic review provides a comprehensive overview of the proteomic biomarkers identified in urogenital cancers, including prostate, bladder, and kidney cancers. The findings highlight the significant potential of these biomarkers in enhancing diagnostic precision, prognostic assessment, and the therapeutic targeting of these malignancies. These biomarkers, derived from different sources such as tissue, blood, and urine, shed light on several biological pathways and processes that could be pivotal in the pathogenesis of these cancers. However, the clinical implementation of these biomarkers requires further validation studies in larger and diverse cohorts to establish their reliability, sensitivity, and specificity. Moreover, the potential for multiplex biomarker panels to offer a more comprehensive understanding of disease complexity and heterogeneity is highlighted. Future research should also take into account the cost-effectiveness of these biomarkers to ensure their broad accessibility and usage in a clinical setting. While this systematic review brings us one step closer to personalized medicine in urogenital cancers, it also underlines the challenges and opportunities that lie ahead in the biomarker discovery journey.

Overall, the field of proteomics holds significant promise for improving our understanding of urogenital cancers. The identified biomarkers in this review represent potential targets for future research aiming to enhance early detection, risk stratification, and treatment of urogenital cancers. However, further in-depth studies are essential to validate these potential biomarkers and to understand their role in the complex biology of cancer. It is hoped that with advancements in proteomic technologies and the combination of proteomic data with other types of data such as genomics and transcriptomics, we will move closer toward precision medicine in urogenital cancers.

## Figures and Tables

**Table 1:** Top 10 enriched pathways in prostate cancer. Pathway analysis of 108 significantly differentially expressed proteins from prostate cancer was carried out using FunRich.

No.	Pathway	Number of Proteins from the Dataset	Proteins from Background Dataset	*p*-Value	FDR *p*-Value	Altered Proteins from the Dataset
1	Smooth muscle contraction	3	24	0.0001	0.1999	CALD1; MYL6; TPM4
2	Muscle contraction	3	50	0.0011	1	CALD1; MYL6; TPM4
3	Epithelial-to-mesenchymal transition	3	185	0.0396	1	PTGDS; CALD1; TAGLN
4	Semaphorin interactions	2	64	0.0283	1	MYL6; HSP90AA1
5	Endosomal sorting complex required for transport (ESCRT)	2	28	0.0058	1	CHMP4C; CHMP2B
6	Membrane trafficking	2	84	0.0466	1	CHMP4C; CHMP2B
7	Integrin family cell surface interactions	7	1375	0.3354	1	TGM2; STMN1; HSP90AA1; BAIAP2; LAMA2; HMGB1; TAGLN
8	IFN-gamma pathway	4	1293	0.8127	1	STMN1; HSP90AA1; BAIAP2; TAGLN
9	Syndecan-1-mediated signaling events	4	1297	0.8148	1	STMN1; HSP90AA1; BAIAP2; TAGLN
10	Regulation of CDC42 activity	3	768	0.6314	1	STMN1; HSP90AA1; BAIAP2

**Table 2:** Top 10 enriched pathways in bladder cancer. Pathway analysis of 143 significantly differentially expressed proteins from bladder cancer was carried out using FunRich.

No.	Pathway	Number of Proteins from the Dataset	Proteins from Background Dataset	*p*-Value	FDR *p*-Value	Altered Proteins from the Dataset
1	Immune system	6	522	0.009438	1	C3; C1R; C6; C7; NRAS; PVR
2	Innate immune system	4	183	0.00398	1	C3; C1R; C6; C7
3	Complement cascade	4	22	0.00000095	0.001587	C3; C1R; C6; C7
4	Beta3 integrin cell surface interactions	3	43	0.000479	0.799287	FGA; LAMA4; PVR
5	Mesenchymal-to-epithelial transition	3	223	0.046173	1	EPS8L2; EPS8L1; S100P
6	Epithelial-to-mesenchymal transition	3	185	0.028762	1	C1R; SERPINF1; MYLK
7	Signaling by FGFR	2	95	0.046495	1	EGFR; NRAS
8	C-MYB transcription factor network	2	84	0.037188	1	NRAS; MPO
9	Endogenous TLR signaling	2	57	0.01807	1	S100A8; S100A9
10	Trk receptor signaling mediated by the MAPK pathway	2	34	0.006683	1	NRAS; EHD4

**Table 3:** Top 10 enriched pathways in kidney cancer. Pathway analysis of 248 significantly differentially expressed proteins from kidney cancer was carried out using FunRich.

No.	Pathway	Number of Proteins from the Dataset	Proteins from Background Dataset	*p*-Value	FDR *p*-Value	Altered Proteins from the Dataset
1	Metabolism of amino acids and derivatives	42	188	1.6903 × 10^−12^	2.819 × 10^−9^	DLST; HSD17B10; ACAT1; HIBADH; BCKDHA; GLUD1; GRHPR; HIBCH; ACADSB; ALDH7A1; OGDH; ALDH6A1; ALDH4A1; GATM; ASS1; AGMAT; FTCD; DDC; AUH; QDPR; BBOX1; GOT2; ALDH9A1; GOT1; HPD; DBT; GCDH; MCCC1; DLD; AASS; MCCC2; SHMT1; BCKDHB; OAT; IVD; HGD; HAAO; MRI1; KYNU; PSMB8; PSMB9; PSME2
2	Metabolism of lipids and lipoproteins	40	257	3.77293 × 10^−7^	0.0006293	HADHA; ACAT1; UGT1A9; DECR1; IDH1; HADH; ECHS1; LRP2; BDH1; PCCB; GK; PCCA; GPD1; ACADM; AMACR; PLIN2; APOA1; HSPG2; CPT2; ACAA1; GGT5; HMGCL; ACOX1; OXCT1; HMGCS2; CUBN; ACSL1; AMN; SLC27A2; MUT; ECI1; CRAT; P4HB; ACLY; SCARB1; PTGES3; HADHB; TXNRD1; ACADS; HSD3B7
3	Fatty acid, triacylglycerol and ketone body metabolism	25	83	7.33774 × 10^−11^	1.224 × 10^−7^	HADHA; ACAT1; UGT1A9; DECR1; HADH; ECHS1; BDH1; PCCB; GK; PCCA; GPD1 ACADM; PLIN2; CPT2; HMGCL; ACOX1; OXCT1; HMGCS2; ACSL1; MUT; ECI1; ACLY; HADHB; TXNRD1; ACADS
4	Pyruvate metabolism and citric acid (TCA) cycle	21	31	3.72184 × 10^−18^	6.208 × 10^−15^	DLST; ACO2; FH; SDHB; PDHB; SUCLG2; OGDH; SUCLG1; IDH2; SDHA; PDHA1; CS; MDH2; DLD; L2HGDH; SUCLA2; NNT; DLAT; PDK2; PDHX; PDK1
5	Fatty acid beta-oxidation I	13	19	9.29191 × 10^−12^	1.55 × 10^−8^	HADHA; HSD17B10; HADH; ECHS1; ACADM; ACAA2; ACAA1; ECI2; EHHADH; ACSL1; SLC27A2; ECI1; HADHB
6	Glucose metabolism	12	36	2.13353 × 10^−6^	0.0035587	PCK2; GOT2; PGK1; MDH2; GOT1; PC; PCK1; SLC25A10; SLC25A11; MDH1; PYGL; TPI1
7	Iron uptake and transport	12	37	2.96762 × 10^−6^	0.00495	ATP6V1E1; ATP6V1A; ATP6V1H; ATP6V1B1; ATP6V1G1; ATP6V1F; ATP6V1B2; ATP6V0A1; ATP6V1C1; ATP6V0D1; HMOX1; TF
8	Mitochondrial fatty acid beta-oxidation	11	14	3.49111 × 10^−11^	5.823 × 10^−8^	HADHA; DECR1; HADH; ECHS1; PCCB; PCCA; ACADM; MUT; ECI1; HADHB; ACADS
9	Gluconeogenesis	11	20	1.10997 × 10^−8^	1.851 × 10^−5^	PCK2; GOT2; PGK1; MDH2; GOT1; PC; PCK1; SLC25A10; SLC25A11; MDH1; TPI1
10	Transferrin endocytosis and recycling	11	27	5.58496 × 10^−7^	0.0009316	ATP6V1E1; ATP6V1A; ATP6V1H; ATP6V1B1; ATP6V1G1; ATP6V1F; ATP6V1B2; ATP6V0A1; ATP6V1C1; ATP6V0D1; TF

## Supplementary Materials

The following supporting information can be downloaded at https://www.mdpi.com/article/10.3390/cancers16010022/s1, Supplementary Figure S1: Comparisons of differentially expressed proteins identified in prostate, bladder, and kidney cancers. Supplementary Figure S2: GO molecular functions for significantly expressed proteins in (A) prostate, (B) bladder, and (3) kidney cancers. Supplementary Figure S3: GO cellular localizations for significantly expressed proteins in (A) prostate, (B) bladder, and (3) kidney cancers. Supplementary Figure S4: Altered pathways (individual) for significantly expressed proteins in (A) prostate, (2) bladder, and (3) kidney cancers. Supplementary Table S1: Summary of annotated biomarkers in prostate, bladder and kidney cancer. Table S2: Complete list of proteins from kidney cancer included in meta-analysis. Combined statistics of these proteins are also depicted.
