# Supplementary material for: Deciphering Urogenital Cancers through Proteomic Biomarkers: A Systematic Review and Meta-Analysis
Source: Cancers (Basel). 2023 Dec 20;16(1):22. doi: 10.3390/cancers16010022 (PMC10778028; doi:10.3390/cancers16010022)
Supplement: Supplementary file 1 [file cancers-16-00022-s001.zip › Supplementary figures.pdf]

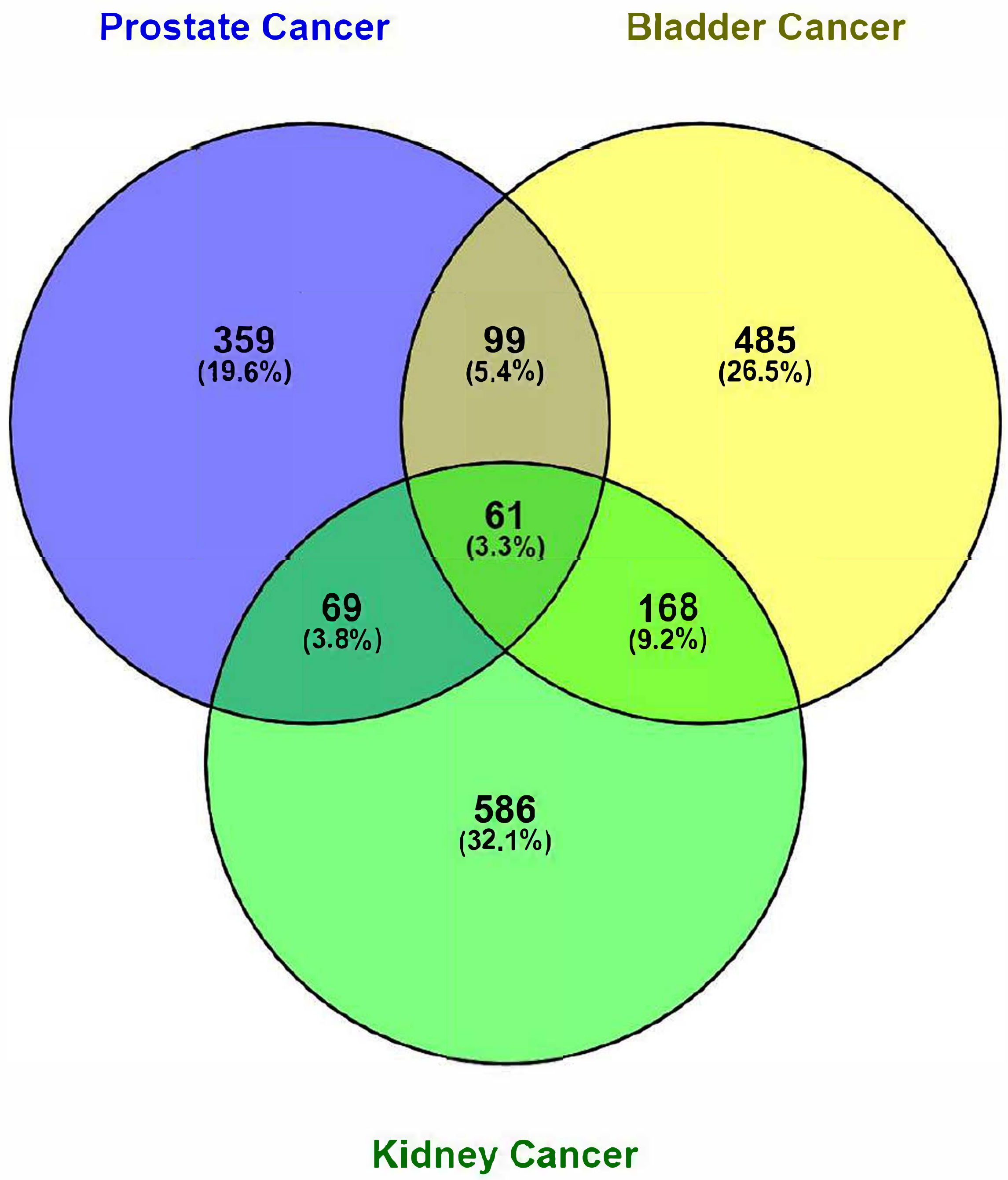

Supplementary Figure S1

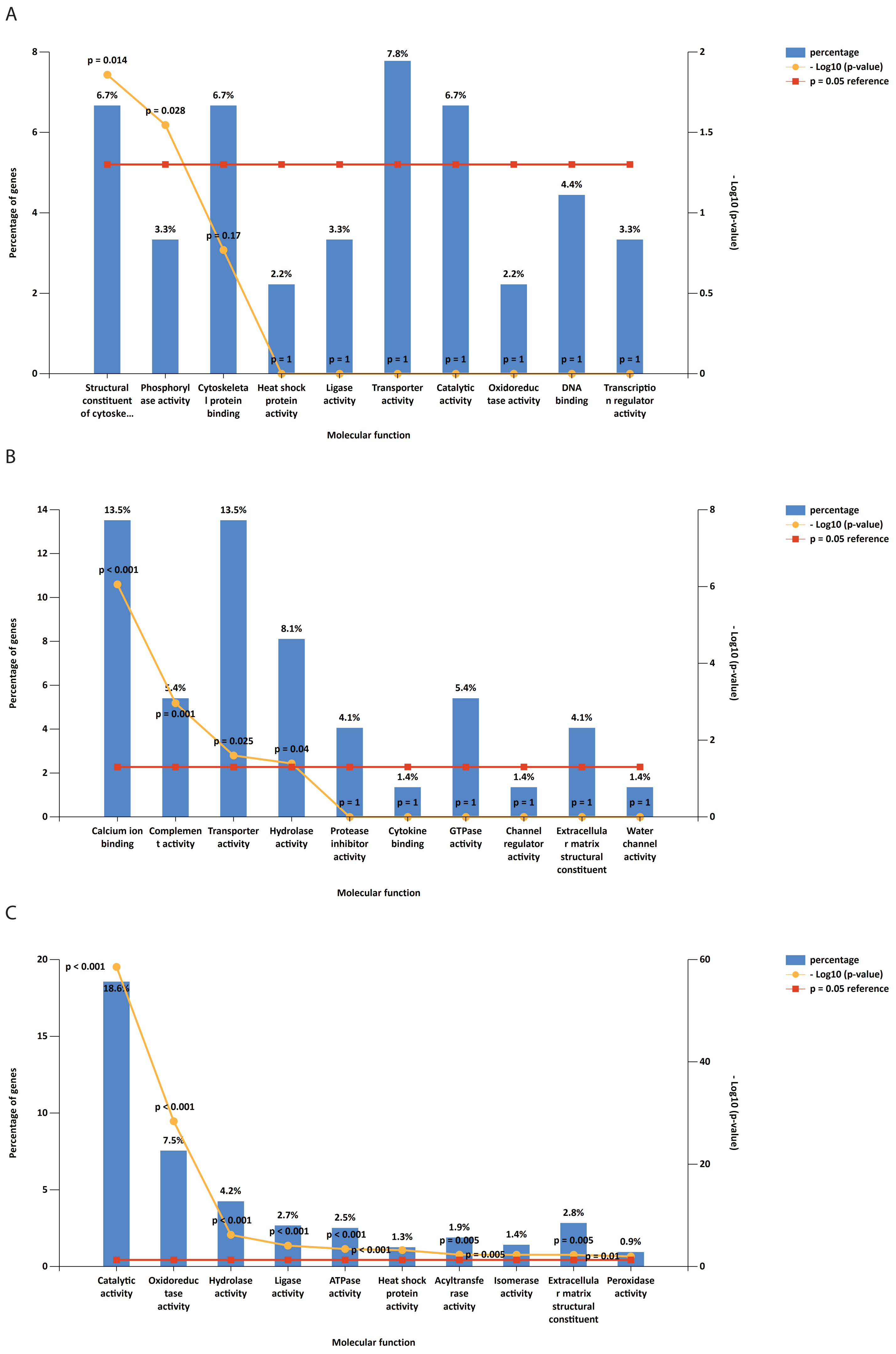

Supplementary Figure S2

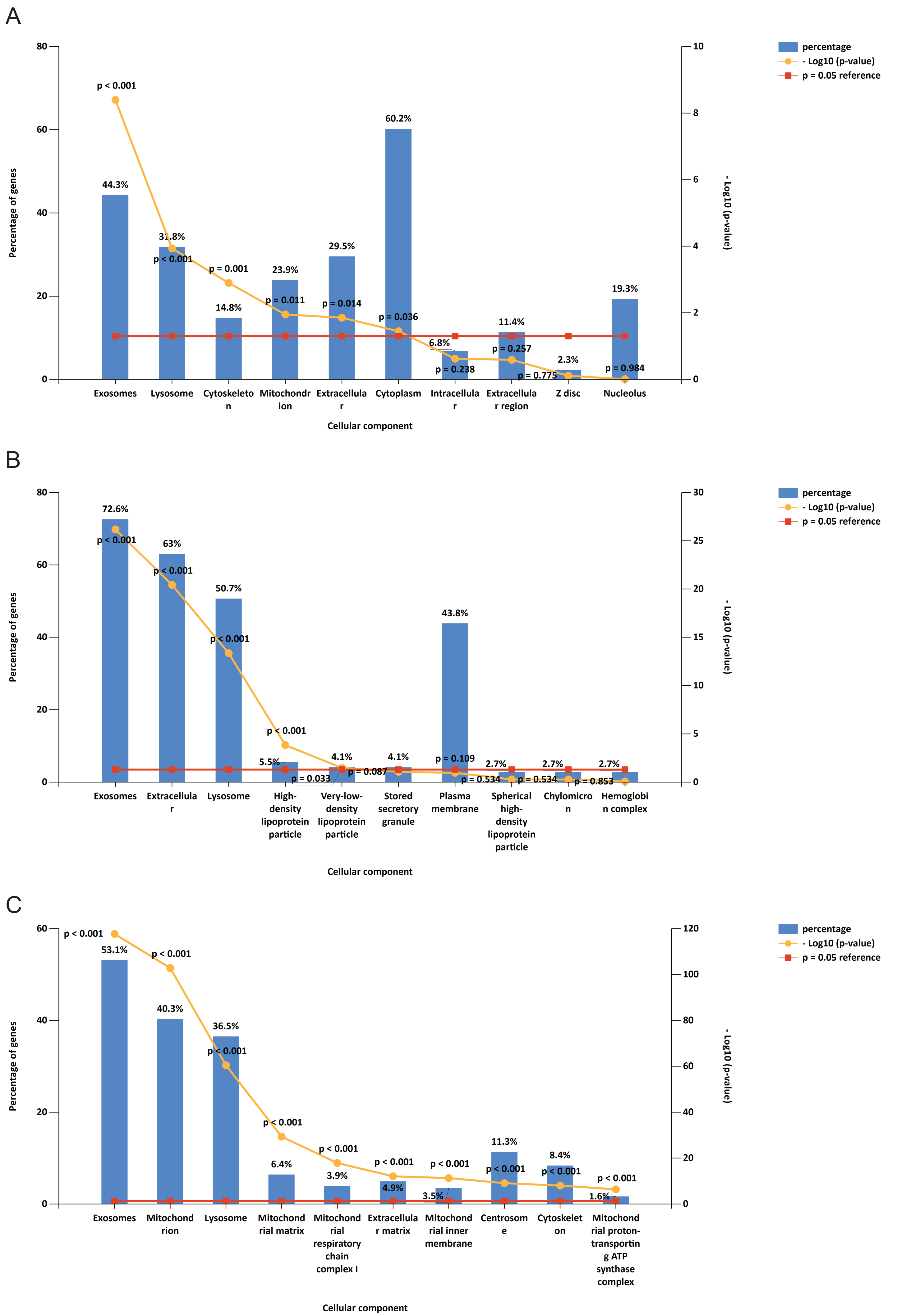

Supplementary Figure S3

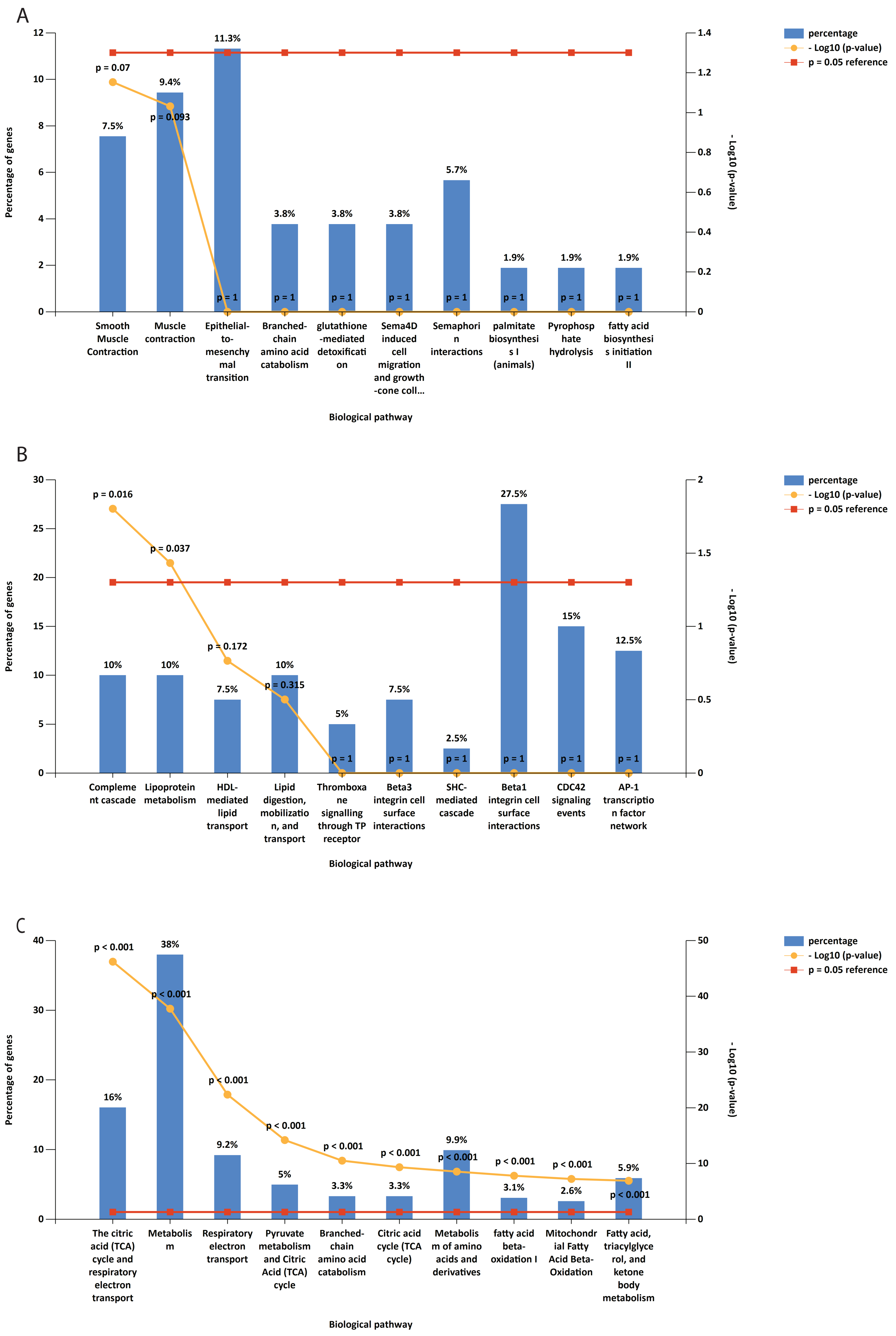

Supplementary Figure S4
